# Computer-assisted rehabilitation of attention in pediatric multiple sclerosis and ADHD patients: a pilot trial

**DOI:** 10.1186/s12883-018-1087-3

**Published:** 2018-06-08

**Authors:** Marta Simone, Rosa Gemma Viterbo, Lucia Margari, Pietro Iaffaldano

**Affiliations:** 1 Child Neuropsychiatry Unit, Department of Basic Medical Sciences, Neurosciences and Sense Organs, University of Bari “Aldo Moro”, Bari, Italy; 2 MS Centre, Department of Basic Medical Sciences, Neurosciences and Sense Organs, University of Bari “Aldo Moro”, Bari, Piazza G. Cesare, 11, 70121 Bari, Italy

**Keywords:** Multiple sclerosis, Attention deficit, Rehabilitation, ADHD

## Abstract

**Background:**

The treatment of cognitive deficits is challenging in pediatric onset multiple sclerosis (POMS) and in patients with attention deficit hyperactivity disorder (ADHD). We performed a pilot double-blind RCT to evaluate the efficacy of a home-based computerized-program for retraining attention in two cohorts of POMS and ADHD patients.

**Methods:**

POMS and ADHD patients failing in at least 2/4 attention tests on a neuropsychological battery were randomized to specific or nonspecific computerized training (ST, nST), performed in one-hour sessions, twice/week for 3 months. The primary outcome was the effect of the training on global neuropsychological performances measured by the cognitive impairment index (CII).

The efficacy of the intervention was evaluated in each disease group by using repeated measures ANOVA.

**Results:**

Sixteen POMS (9 females, age 15.75 ± 1.74 years) and 20 ADHD (2 females, age 11.19 ± 2.49 years) patients were enrolled. In POMS patients the ST exposure was associated to a significantly more pronounced improvement of the CII (*p* < 0.0001) and on cognitive test exploring attention, concentration, planning strategies and visuo-spatial memory performances in comparison to nST exposure. In ADHD patients the difference between the ST and nST on the CII was not statistical significant (*p* = 0.06), but a greater effect of the ST was found only on cognitive test exploring attention and delayed recall of visuo-spatial memory performances.

**Conclusions:**

Our data suggest that a cognitive rehabilitation program that targets attention is a suitable tool for improving global cognitive functioning in POMS patients, whereas it has a less pronounced transfer effect in ADHD patients.

**Trial Registration:**

ClinicalTrials.gov; NCT03190902; registration date: June 15, 2017; retrospectively registered.

## Background

The presence of cognitive deficits of varied intensity is a characteristic of psychiatric disorders of childhood and adolescence such as Attention Deficit Hyperactivity Disorder (ADHD), but also of neurological pathologies such as pediatric onset multiple sclerosis (POMS).

ADHD is one of the most common neurodevelopmental disorders characterized by pervasive patterns of inattention and/or impulsivity/hyperactivity and a range of cognitive dysfunctions that often persist into adulthood [1, 2].

POMS represent 5–10% of total MS population [3]. Cognitive dysfunction is one of the most remarkable features of MS and particularly in POMS. The percentage of patients with POMS with at least a mild cognitive deficit ranges from 30 to 50% [4–9]. The most affected cognitive domains in POMS are complex attention, information processing speed, executive functions, verbal and visual memory, reasoning and problem solving [4, 9]. Longitudinal studies from US and Canada groups showed a cognitive stability in POMS patients over time [10, 11]. The US study reported an overall percent of patients with cognitive impairment, defined as having one-third or more test scores in the impaired range, of 37.3% at baseline and 32.3% after a mean follow-up of 1.64 year [10]. A Canadian study, examining patients and controls over a 1-year period found that controls generally showed greater improvement than patients, and 25% of patients showed clinically significant decline [11].

In contrast, an Italian longitudinal study demonstrated that cognitive impairment in POMS tend to worsen after a mean period of 2 years since baseline evaluation [12]. At follow-up 75% of the cases were classified as having a deteriorating cognitive performance. Changes were prominent in tests of verbal memory, complex attention, verbal fluency, and receptive language. However the same group reversed their initial negative findings showing that after 5 years from baseline there was more stability than decline [13].

Interestingly, a variety of psychiatric symptoms can occur in POMS. Approximately one-third of children suffer from depressive symptoms and one-fourth of POMS report fatigue [12, 14]. Moreover, one study has reported ADHD as one of the most frequent comorbid psychiatric disorder in POMS [15]. The functional consequences of cognitive impairment can be particularly striking in children and adolescents, since they occur during their formative years, therefore it may affect their academic and social activities. Cognitive training during the developmental age when brain plasticity is at the highest expression can induce a strengthening of the key brain networks implicated in POMS and ADHD.

In adult onset MS patients several studies examined the efficacy of cognitive rehabilitation programs related to attention and additional cognitive domains [16, 17]. Some of them provided class I evidence of beneficial effects of these training programs through randomized clinical trials (RCTS) [18–20].

To date the efficacy of specific cognitive rehabilitation interventions has never been evaluated by a RCT in POMS. Whereas, the fMRI effect of a working memory training has been reported in a small case series of 5 juvenile MS patients [21]. Conversely, in the last years, several RCTs [22–25] assessed the efficacy of cognitive training as a potential non-drug alternative treatment for ADHD disorder [26]. Most of the cognitive trainings focused on the working memory or attention dysfunctions. Preliminary evidence suggests that cognitive remediation might be at least partially effective in the ADHD treatment [27–32]. It is argued that cognitive training can potentially reduce ADHD symptoms and might improve functioning by targeting neuropsychological deficits thought to mediate ADHD pathophysiology [33–37].

In this exploratory pilot study, we assessed by a double-blind RCT the efficacy of a home-based computerized program for retraining attention dysfunction in two cohorts of POMS and ADHD patients. The results in POMS were compared to those obtained in ADHD patients.

## Methods

### Standard protocol approval, patient consent and recruitment

The study was conducted with approval of the institutional review board (Comitato Etico Indipendente Azienda Ospedaliero-Universitaria Consorziale Policlinico - Approval Number: 0070059/CE). Parents of the participants signed an informed consent.

Recruitment (September–December 2015, predetermined) and assessments were performed at the MS Centers and at the Child Neuropsychiatry Unit both of the University of Bari. Due to the monocentric nature of the study and due to the absence of external funding resources the recruitment has been limited only to referrals to the these two centers. Clinical trial registration information: ClinicalTrials.gov number NCT03190902. Clinical trial registration date: June 15, 2017.

### Study population

#### POMS

We recruited POMS outpatients consecutively referred to the MS Centers at the University of Bari who met the inclusion/exclusion criteria during the study period. Inclusion criteria were: POMS diagnosed according to the most recent diagnostic criteria [38], aged < 18 years, with an Expanded Disability Status Scale (EDSS) score ≤ 5.5, impairment on at least 2/4 attention tests (see below) defined as scores < 1.5 standard deviation (SD) of normative values [9, 12, 13]. To facilitate recruitment we excluded only patients with important impairments on other cognitive tasks, defined as performance ≤2.0 SD of normative values, still including subjects with milder degrees of impairment (e.g. cognitive scores between 1.51 and 1.99 SD below the normative values). Exclusion criteria were: severe visual loss (unable to read Times New Roman font 16 with the best correction), major psychiatric illness (any severe disabling psychiatric disorders [i.e. major depression, obsessive-compulsive disorder, psychotic disorder] which could interfere with: the understanding of the protocol and of the informed consent; the overall engagement in the study and in particular with the adherence with the treatment regimen and the compliance with the study visits and procedures), alcohol or substance abuse, education < 5 years, previous cognitive rehabilitation training, ongoing relapse or steroid treatment during the 30 days preceding enrollment. Disease-modifying treatments (DMTs) and symptomatic treatments were maintained unchanged during the study.

#### ADHD

We enrolled ADHD outpatients consecutively referred to the Child Neuropsychiatry Unit at the University of Bari. We included only ADHD patients with the subtype inattention, not previously exposed or not treated with any psychotropic drug. ADHD diagnosis was performed according the DSM-5 criteria and the NIMH Collaborative Multisite Multimodal Treatment Study of Children With Attention- Deficit/Hyperactivity Disorder (MTA) - Swanson, Nolan, and Pelham IV Rating Scale (MTA-SNAP-IV), Conner’s Parent Rating Scale Revised (CPRS-R), Conner’s Teacher Rating Scale Revised (CTRS-R), Child Behavior Checklist (CBCL), Kiddie Schedule for Affective Disorder and Schizophrenia (K-SADS). The same inclusion/exclusion criteria referring the cognitive performances were applied to the ADHD patients.

#### Study procedures

We applied the same study procedures already reported in a previous study which evaluate the effect of the cognitive rehabilitation program in adult-onset MS patients [18]. Patients were randomized to receive a specific computer training (ST) or to receive a nonspecific computer training (n-ST) with a 1:1 ratio. Randomization was performed by an independent researcher on the basis of a computerized list of random numbers. A psychologist, blind to the study, was responsible for administering and evaluating the neuropsychological tests, whereas an independent researcher, who was not blind to the study, was responsible for setting up the ST and n-ST programs, explaining the training procedure and supervising the training program.

As defined by the study protocol, during the study period, in case of a suspected MS relapse, the patient underwent a neurological examination within 48 h and a standard steroid treatment was prescribed if needed. A confirmed MS relapse was a reason for study discontinuation.

#### Study design

This is a single-centre, parallel group double blind-RCT.

### Assessment and outcome measures

At the baseline visit, a neurologist and a child/adolescent neuropsychiatrist collected demographic and clinical information of POMS and ADHD (familiar background, any past medical history) patients.

The handedness was evaluated in both groups by the Edinburgh Inventory [39].

The neuropsychological assessment was performed by a psychologist. Two alternate versions of the tests were used at different assessment points.

The primary outcome was to evaluate the effect of the cognitive training on neuropsychological performances.

The neuropsychological test battery assessed the following cognitive areas:

- Verbal learning and delayed recall: Selective Reminding Test (SRT) and Selective Reminding Test–Delayed (SRT-D) from the Rao Brief Repeatable Battery (BRB) [40];
- Visuo-spatial learning and delayed recall: Spatial Recall Test (SPART) and Spatial Recall Test–Delayed (SPART-D) from the BRB [40];
- Concentration, attention, processing speed, working memory and cognitive flexibility: Symbol Digit Modalities Test (SDMT) from the BRB and the Trail Making Tests (TMT) A and B [41];
- Expressive language: Semantic Verbal Fluency Test (SVFT), in which the subject is asked to produce as many words as possible belonging to a semantic category (colors, animals, fruits, cities) within 120 s. The score is the average of correct words [42].
- Planning: Tower of London Test (TOL). The initial and target configuration of the TOL were presented under the form of 2 identical kits made of a wooden base (22 16 1 2 cm) with 3 rods of 12, 8, and 4.5 cm, and 3 balls (yellow, red, and blue) of 3 cm in diameter. The subject was required to obtain the target configuration in a minimum number of moves, according to the following rules: move only 1 ball at a time; place at most 1 ball on the shortest peg and 2 balls on the middle one; move each ball only from one peg to another. There were no time limits. The execution time and the number of moves were recorded by the examiner. The results (target configuration attained or not, abandoned) and any rule violations were noted [43].
- Depression: self-assessed by patients through the Children’s Depression Inventory (CDI) with the assistance of the psychologist [44].
- Kiddie Schedule for Affective Disorder and Schizophrenia (K-SADS).

The psychologist administered the above battery, using alternative versions of the tests, at baseline, and within 1 week following the end of the training program. At the same time intervals, self-assessed measures were also repeated.

### Intervention

The ST was based on the Attention Processing Training program (APT) [45]. This program targets focused, sustained, selective, alternating and divided attention and consists of a group of hierarchically organized tasks that exercise different components of attention, proceeding from sustained to selective, alternating and finally divided attention exercises. The sequence of the exercises places increasing demands on complex attention control and working memory systems (e.g. identification of target numbers or letters in the presence of distracter images and noises).

The n-ST consisted of a series of nonspecific exercises including the following: text reading and comprehension: e.g. reading brief text extracted from novels, journals, newspaper (without requesting a feedback nor verbal nor written); give feedback on proverbs comprehension; description of pictures: e.g. try to provide a brief verbal description of simple pictures; enumerating words classified in different categories; trying to provide at least 3 synonyms for a given list of words; etc.

Before starting the training at home, a psychologist who was not blind to the patient assignment group conducted a training session for each patient at the MS Clinic, in the presence of the caregiver/parent, in which instructions and procedures for the use of the ST and n-ST were provided (Patients and their parents were not aware about the treatment arm assigned). Each patient was treated at home twice a week for three consecutive months. Each training session lasted 1 h. Each patient applied the training on his/her own under the supervision of the caregiver/parent, who also collected and stored forms reporting patient performance during each training session. During the study period, the psychologist called patients every week and met patients and their caregiver/parent every month to check patient compliance and possible difficulties in the use of the training program.

### Statistical analysis

Given the exploratory nature of this pilot trial, no sample size analysis was performed. Continuous variables were described as mean and standard deviation (SD), categorical variables as frequency and percentage. Group comparison has been performed using the Student’s t test, the Mann-Whitney U test and the Fisher’s exact test when appropriate.

A global score, defined Cognitive Impairment Index (CII), allowing the evaluation of changes in cognitive performances independently by the number of cognitive tests failed at the neuropsychological evaluation, was obtained using the mean and SD from the normative values for each test [13, 46–48].

For each patient, a grading system was applied to individual cognitive tests, based on the number of SDs below the control mean (i.e. grade 0 was given if the patient scored at or above the control mean, 1 if he/she scored below the control mean, but at or above 1 SD below the control mean, and so on until all patient scores were accommodated) [13, 46–48]. Finally, all the patient’s scores were summed to give one overall measure of cognitive function.

The efficacy of ST on the global cognitive functioning, measured by the CII, and on performance of each cognitive test was evaluated in each disease group by using a 2 (Group–ST and n-ST) × 2 (Time–baseline, 3 months) mixed factorial design, with repeated measures on the second factor.

Finally, the comparative efficacy of the ST on the global neuropsychological performances between the 2 disease groups was assessed by using a generalized linear model. In this model the estimated mean difference between performances recorded after training and those recorded before training on the CII for each disease group was compared. Age, sex and school education were included as covariates in this model.

Statistical analysis was performed by using SPSS software (SPSS, version 22.0; SPSS, Chicago, Ill).

## Results

**Fig. 1:**
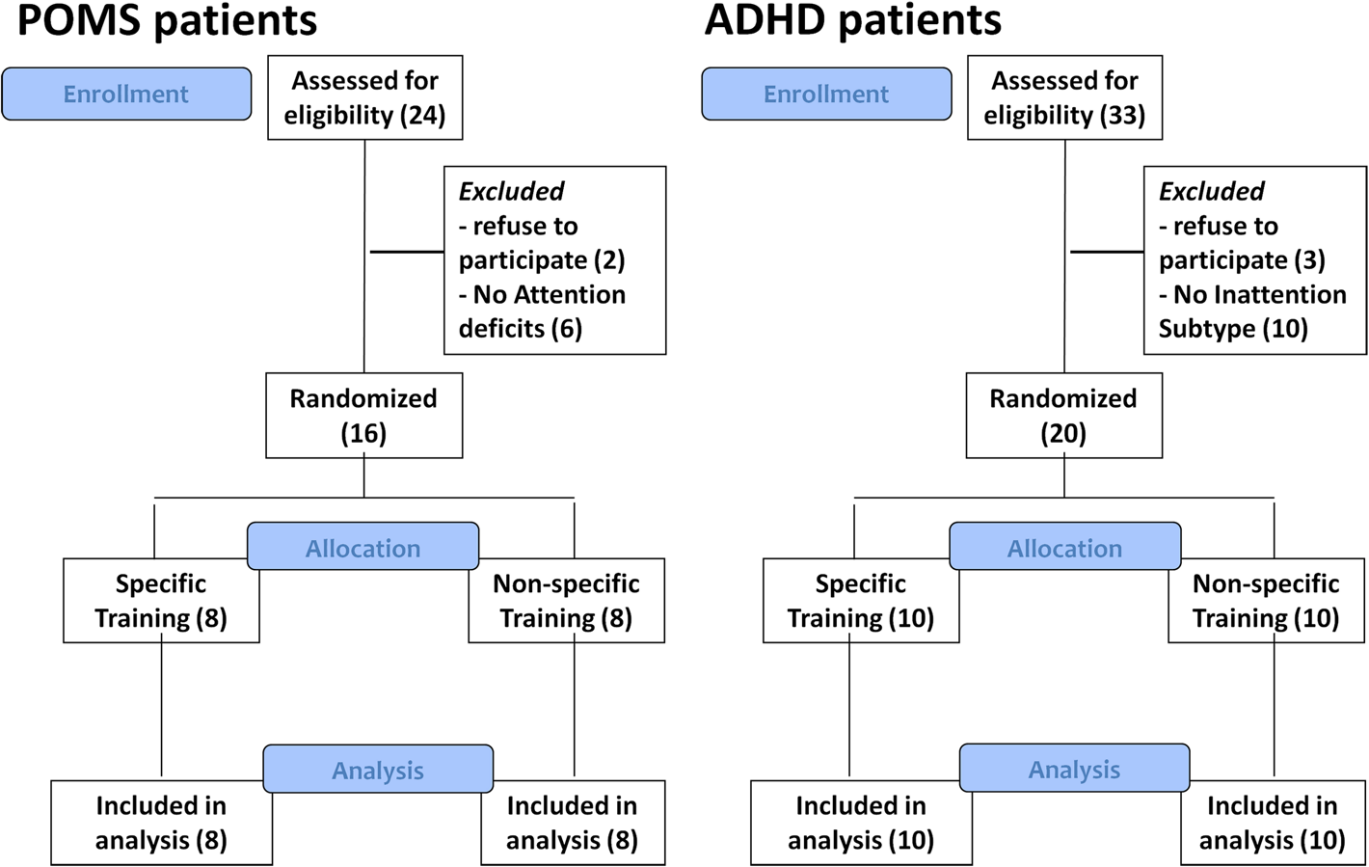
CONSORT 2010 Flow Diagram

Twenty-four POMS and 33 ADHD patients were assessed for eligibility during the recruitment period. In the POMS group, 2 patients refused to participate, 6 patients did not meet the inclusion criteria regarding the presence of deficits on at least 2/4 attention performance tests. In the ADHD group, 3 patients refused to participate, 10 patients were excluded because they were affected by a combined ADHD. Finally, 16 POMS and 20 ADHD patients were enrolled. All the patients enrolled completed the study procedures and assessments. No patients with POMS reported relapses during the study period. There were no drop-out (Fig. 1).

The comparison of the baseline cognitive performances between the 2 disease conditions is reported in Table 1. ADHD patients showed significantly worse attention, concentration, processing speed, working memory and cognitive flexibility performances at baseline as measured by the SDMT, TMT-A and TMT-B (*p* < 0.05) in comparison to POMS patients.

**Table 1:** Comparison of baseline neuropsychological performances in POMS and ADHD

Cognitive test	POMS	ADHD	*P* value
SRT- LTS	27.3 (10.1); 24 (9–52)	30.9 (6.8); 28.5 (20–44)	0.1
SRT – CLTR	21.3 (9.1); 21.5 (2–40)	21.9 (8.6); 21 (7–44)	1.0
SPART	21.0 (3.8); 22 (15–25)	19.8 (4.6); 20 (13–30)	0.2
SDMT	22.5 (4.5); 23 (16–29)	17.3 (8.5); 14.5 (8–43)	0.002
Trail Making Test A^a^	37.0 (10.6); 37.5 (21–58)	54.0 (23.1); 57 (8–100)	0.02
Trail Making Test B^a^	108.1 (68.6); 97.5 (0–233)	182.7 (64.9); 188 (96–333)	0.004
SRT-D	6.0 (2.2); 6 (0–9)	6.7 (2.0); 7 (3–11)	0.5
SPART-D	6.9 (1.2); 7 (5–10)	6.5 (2.3); 5.5 (4–10)	0.2
Tower of London^a^	15.7 (5.8); 14 (10–31)	14.7 (10.1); 14 (0–33)	0.7
Cognitive Impairment Index	22.4 (3.1); 22 (19–29)	23.6 (4.2); 24 (16–30)	0.3
CDI	12.9 (6.9); 15 (0–25)	13.4 (7.0); 14.5 (2–25)	1.0

Data are reported as mean (SD), Median (min - max)

*Abbreviations*: *POMS* pediatric onset multiple sclerosis, *ADHD* Attention Deficit Hyperactivity Disorder, *ST* specific training, *nST* non specific training, *SRT-LTS* Selective Reminding Test Long Term Storage, *SRT-CTLR* Selective Reminding Test – Consistent Long-Term Retrieval, *SRT-D* Selective Reminding Test–Delayed, *SPART* Spatial Recall Test, *SPART-D* Spatial Recall Test–Delayed, *SDMT* Symbol Digit Modalities Test, *CDI* Children’s Depression Inventory

^a^Unit of measure: time in seconds

Comparisons of baseline demographic, clinical characteristics, neuropsychological performances (NP) of POMS, and ADHD subgroups underwent ST and n-ST are reported in Tables 2 and 3, respectively. At baseline, no differences were found between the 2 treatment arms regarding sex, age, and in terms of NP performances. The mean ± SD, median (min-max) age, disease duration and EDSS score of the entire POMS cohort were: 15.8 ± 1.7 years, 16.4 (12.4–17.9); 3.4 ± 3.0 years, 2.4 (0.1–10.5); 2.3 ± 0.9 EDSS score, 2.25 (1–3.5), respectively. All POMS patients were under stable (at least 6 months) first line DMTs. The mean ± SD, median (min-max) age of the entire ADHD cohort were: 11.2 ± 2.5 years, 11.7 (7.4–17.6). No ADHD patients received psychoactive medications during the study period.

**Table 2:** Baseline demographic and clinical characteristics of POMS and ADHD subgroups underwent specific and non specific training

POMS			
Variable	Specific training (*n* = 8)	Non specific training (*n* = 8)	*p* - value (t, U, or Fisher’s exact test)
Sex (F/M)	5/3	4/4	1.0
Age, years	15.8 (2.0)	15.7 (1.5)	1.0
Disease Duration, years	3.5 (3.5)	3.3 (2.6)	0.96
Handedness, n. right-handed (%)	7 (87.5)	8 (100)	0.97
Disease modifying therapy, n			
Nothing	2	2	0.67
Interferon beta	6	4	
Glatiramer Acetate	0	1	
Natalizumab	0	1	
Annualized Relapse Rate	0.4 (0.5)	0.3 (0.5)	0.72
EDSS, median (min - max)	2.0 (1.0–3.5)	3.0 (1.0–3.5)	0.28
ADHD			
Variable	Specific training (*n* = 10)	Non specific training (*n* = 10)	
Sex (F/M)	0/10	2/8	0.47
Age, years	11.5 (3.0)	11.3 (2.0)	0.58
Handedness, n. right-handed (%)	9 (90)	9 (90)	1.0

*Abbreviations*: *POMS* pediatric onset multiple sclerosis, *EDSS* Expanded Disability Status Scale, *ADHD* Attention Deficit Hyperactivity Disorder

**Table 3:** Baseline neuropsychological performances in POMS and ADHD subgroups underwent specific (ST) and non specific (nST) trainings

POMS			
Cognitive test	ST	nST	*P* value
SRT- LTS	29.9 (12.6)	24.6 (6.5)	0.2
SRT - CLTR	22.1 (11.0)	20.4 (7.5)	0.6
SPART	19.3 (4.4)	22.8 (2.0)	0.1
SDMT	24.5 (4.6)	20.5 (3.6)	0.1
Trail Making Test A	39.4 (11.5)	34.6 (9.8)	0.5
Trail Making Test B	108.4 (61.4)	107.9 (79.4)	1.0
SRT-D	6.3 (2.8)	5.8 (1.5)	0.2
SPART-D	6.8 (1.0)	7.0 (1.4)	1.0
Tower of London	15.8 (5.4)	15.6 (6.6)	0.8
Cognitive Impairment Index	22.5 (3.9)	22.3 (2.4)	0.9
CDI	14.5 (7.6)	11.4 (6.3)	0.4
ADHD			
Cognitive test	ST	nST	P
SRT- LTS	32.2 (6.9)	29.6 (6.7)	0.4
SRT - CLTR	25.5 (8.2)	18.3 (7.8)	0.1
SPART	18.3 (4.9)	21.2 (4.2)	0.1
SDMT	17.1 (5.5)	17.4 (11.1)	0.4
Trail Making Test A	61.9 (17.3)	46.1 (26.2)	0.1
Trail Making Test B	160.8 (45.4)	204.6 (75.9)	0.3
SRT-D	7.1 (2.1)	6.2 (1.8)	0.6
SPART-D	6.0 (2.2)	6.9 (2.3)	0.2
Tower of London	15.9 (9.1)	13.5 (11.3)	0.5
Cognitive Impairment Index	23.8 (4.7)	23.4 (4.0)	0.8
CDI	13.5 (7.6)	13.2 (6.7)	0.9

*Abbreviations*: *POMS* pediatric onset multiple sclerosis, *ADHD* Attention Deficit Hyperactivity Disorder, *ST* specific training, *nST* non specific training, *SRT-LTS* Selective Reminding Test Long Term Storage, *SRT-CTLR* Selective Reminding Test – Consistent Long-Term Retrieval, *SRT-D* Selective Reminding Test–Delayed, *SPART* Spatial Recall Test, *SPART-D* Spatial Recall Test–Delayed, *SDMT* Symbol Digit Modalities Test, *CDI* Children’s Depression Inventory

### Cognitive performances in POMS

The NP before and after the cognitive training are reported in Table 4.

**Table 4:** Impact of 3-month ST and nST training on cognitive performances in POMS and ADHD groups

	ST		nST		*p*-value
Cognitive Test	Baseline	Post - Treatment	Baseline	Post - Treatment	Effect for group × time
POMS					
SRT- LTS	29.9 (12.6)	35.4 (5.0)	24.6 (6.5)	28.9 (4.2)	0.72
SRT - CLTR	22.1 (11.0)	27.3 (5.2)	20.4 (7.5)	22.6 (6.1)*	0.30
SPART	19.3 (4.4)	25.5 (1.7)*	22.8 (2.0)	23.1 (1.9)	0.004
SDMT	24.5 (4.6)	46.3 (6.7)*	20.5 (3.6)	20.8 (4.1)	< 0.0001
Trail Making Test A	39.4 (11.5)	31.8 (6.6)	34.6 (9.8)	43.8 (10.2)	0.01
Trail Making Test B	108.4 (61.4)	70.5 (32.1)	107.9 (79.4)	64.0 (61.3)	0.87
SRT-D	6.3 (2.8)	8.1 (0.8)	5.8 (1.5)	6.4 (0.7)	0.28
SPART-D	6.8 (1.0)	8.1 (0.8)*	7.0 (1.4)	6.1 (0.8)	0.004
Tower of London	15.8 (5.4)	30.4 (2.5)*	15.6 (6.6)	13.8 (2.0)	< 0.0001
Cognitive Impairment Index	22.5 (3.9)	10.4 (3.3)*	22.3 (2.4)	19.3 (2.2)*	< 0.0001
ADHD					
SRT- LTS	32.2 (6.9)	34.9 (7.3)	29.6 (6.7)	30.5 (6.8)	0.50
SRT - CLTR	25.5 (8.2)	27.3 (5.0)	18.3 (7.8)	22.6 (4.4)	0.51
SPART	18.3 (4.9)	22.0 (4.2)*	21.2 (4.2)	21.2 (3.7)	0.06
SDMT	17.1 (5.5)	35.8 (7.2)*	17.4 (11.1)	22.1 (11.4)	0.004
Trail Making Test A	61.9 (17.3)	38.4 (22.2)*	46.1 (26.2)	31.5 (16.9)	0.51
Trail Making Test B	160.8 (45.4)	108.7 (61.0)*	204.6 (75.9)	178.0 (131.11)	0.51
SRT-D	7.1 (2.1)	6.8 (2.0)	6.2 (1.8)	6.6 (1.0)	0.41
SPART-D	6.0 (2.2)	7.7 (2.0)*	6.9 (2.3)	6.6 (1.2)	0.04
Tower of London	15.9 (9.1)	30.5 (11.0)*	13.5 (11.3)	17.9 (12.6)	0.27
Cognitive Impairment Index	23.8 (4.7)	15.3 (5.6)*	23.4 (4.0)	19.3 (3.6)*	0.06

*Abbreviations*: *POMS* pediatric onset multiple sclerosis, *ADHD* Attention Deficit Hyperactivity Disorder, *ST* specific training, *nST* non specific training, *SRT-LTS* Selective Reminding Test Long Term Storage, *SRT-CTLR* Selective Reminding Test Consistent Long-Term Retrieval, *SRT-D* Selective Reminding Test–Delayed, *SPART* Spatial Recall Test, *SPART-D* Spatial Recall Test–Delayed, *SDMT* Symbol Digit Modalities Test, *CDI* Children’s Depression Inventory

*Indicate significant Effect for time (Baseline vs Post – Treatment comparison); *p* < 0.05

**Fig. 2:**
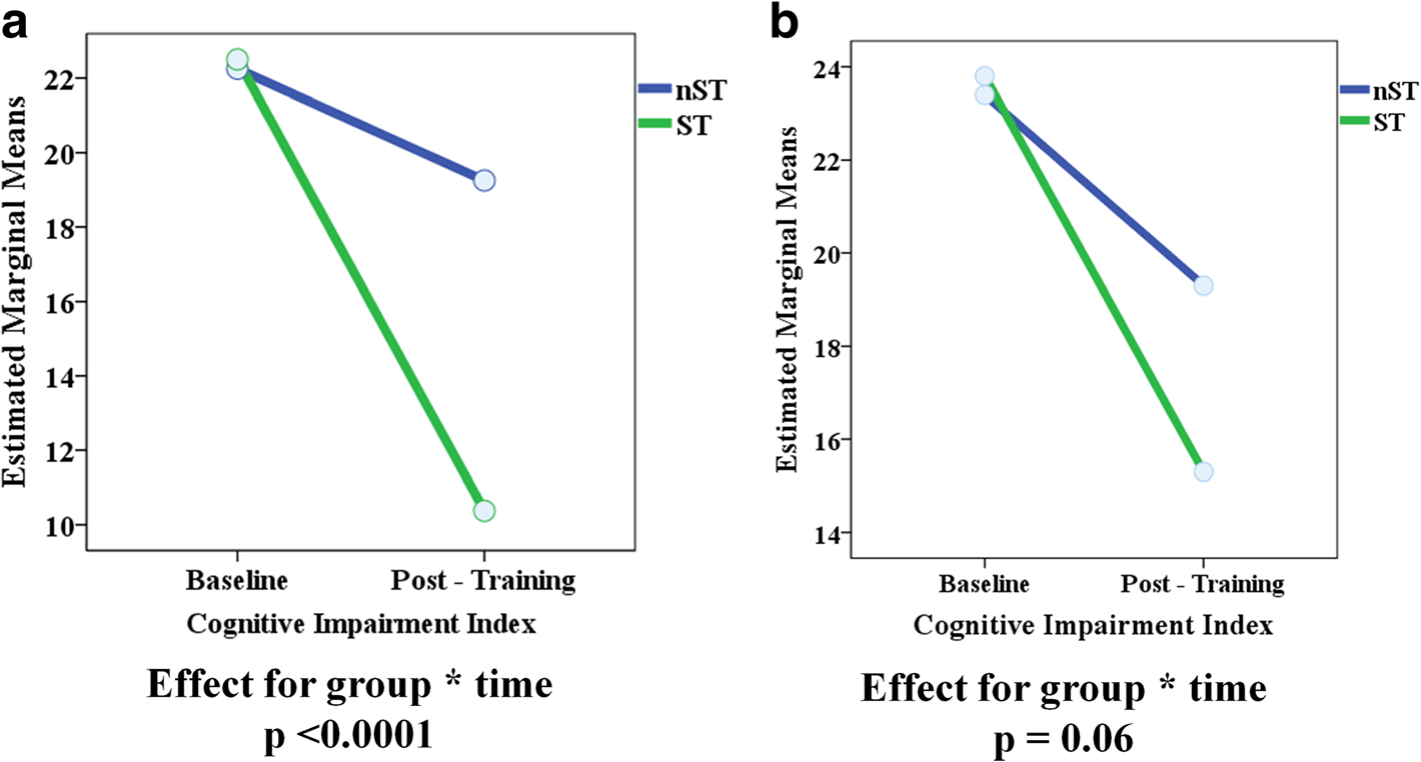
Cognitive Impairment Index before and after the cognitive specific training (ST) and non specific training (nST) in POMS (**a**) and ADHD (**b**)

**Fig. 3:**
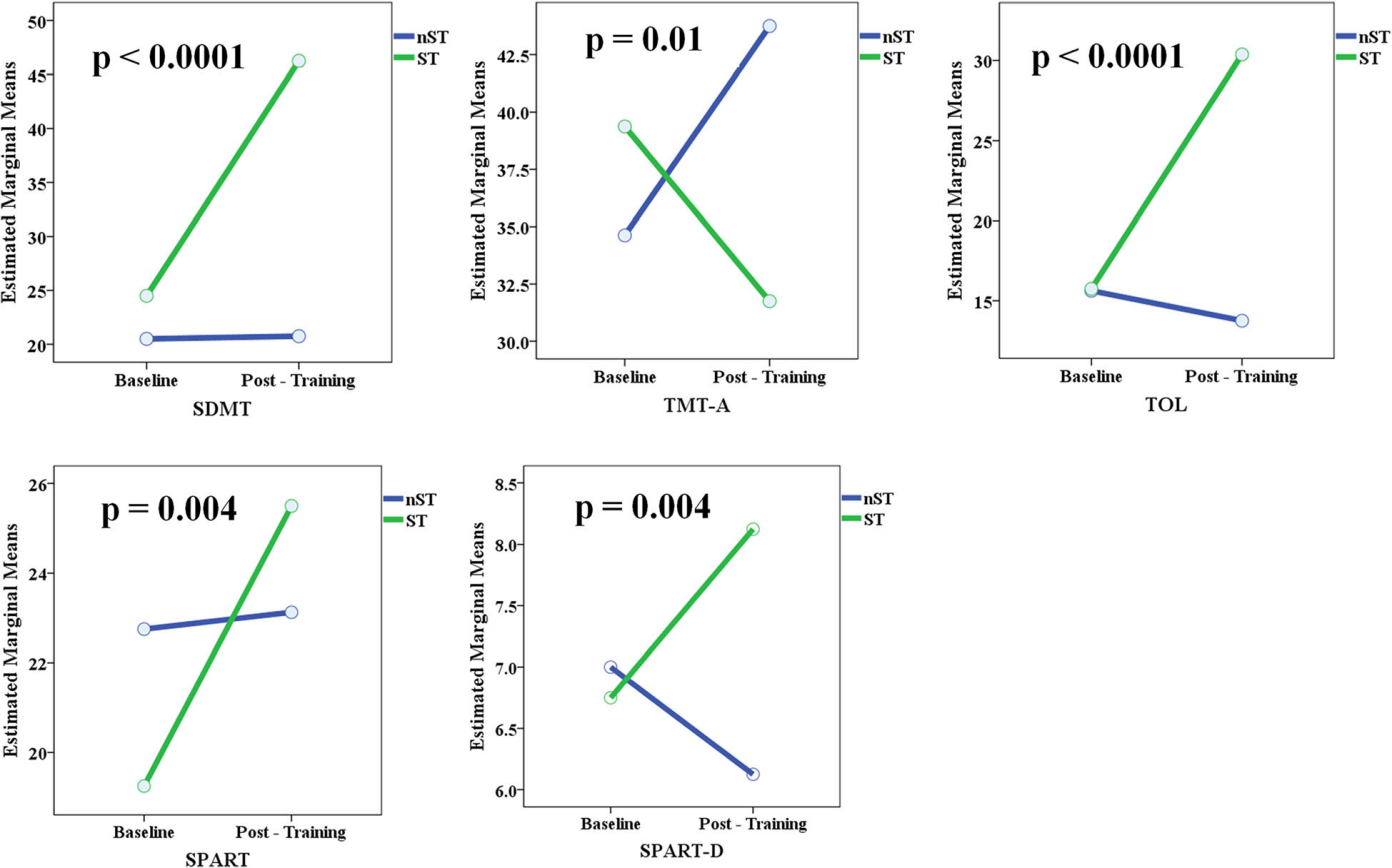
Effect of the cognitive specific training (ST) and non specific training (nST) on SDMT, TMT-A, TOL, SPART, SPART-D performances in POMS patients. P values indicate the effect for group * time interaction

After the 3-month cognitive training, the ST exposure was associated to a significantly more pronounced reduction of the CII in comparison to the nST exposure (*p* < 0.0001) (Table 4 and Fig. 2a). POMS patients treated with ST had a significant higher improvement in their performances on SDMT (p < 0.0001), TOL (p < 0.0001), TMT-A (*p* = 0.01), SPART (*p* = 0.004) and SPART-D (p = 0.004) in comparison to those treated with nST (Table 4 and Fig. 3).

### Cognitive performances in ADHD

**Fig. 4:**
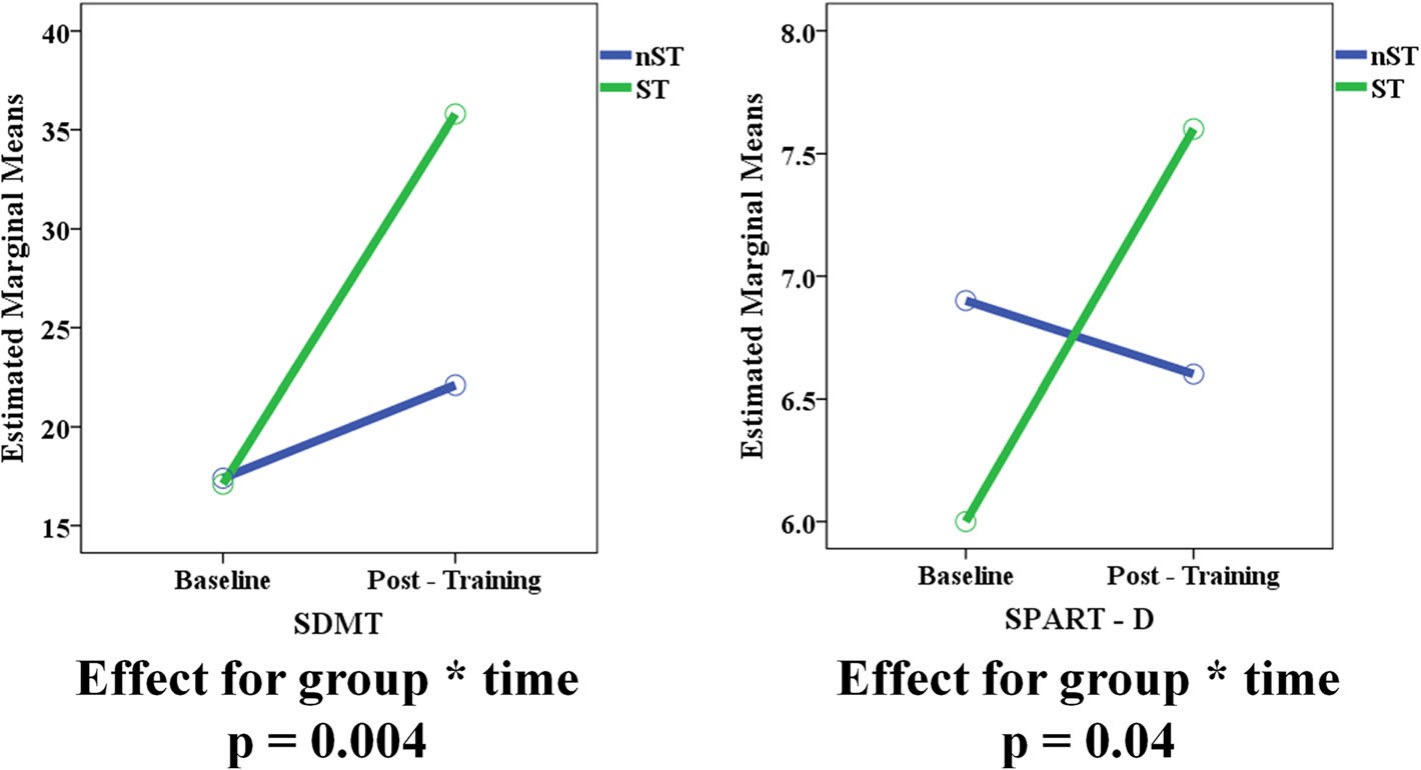
Effect of the cognitive specific training (ST) and non specific training (nST) on SDMT and SPART-D performances in ADHD patients

Unlike in POMS, the difference between the ST and nST subgroups in ADHD patients on the global neuropsychological performances measured by the CII did not reach statistical significance (*p* = 0.06) (Table 4 and Fig. 2b). A greater effect of the ST in comparison to the nST in ADHD patients was found only for performances on SDMT (p = 0.004), and SPART-D (*p* = 0.04) (Table 4 and Fig. 4). No differences were found between the two treatment arms in other cognitive domains.

The generalized linear model performed to evaluate the magnitude of the effect of the ST in both the disease groups demonstrated a significant greater reduction of the CII in POMS than that observed in ADHD patients (*p* = 0.042).

## Discussion

This pilot study confirms the presence of a cognitive impairment in children affected by ADHD and POMS, with a more severe deficit of attention, concentration, processing speed, working memory and cognitive flexibility performances in ADHD than POMS.

Most importantly, the results of this controlled, double-blind, randomized study, demonstrate that a home-based computerized training of specific aspects of attention, the APT, has a different effect on cognitive functions in POMS and ADHD patients.

In POMS, APT improves in the short-term the global cognitive functions and individual performances in several cognitive domains. POMS patients improved in SDMT and TMT-A performances, which evaluate concentration, attention, processing speed, working memory and cognitive flexibility.

Furthermore, we observed an improvement also in cognitive domains not specifically trained by the program. Patients with POMS improve their executive functioning, planning strategies, visuo-spatial memory and delayed recall performances, as assessed by TOL, SPART and SPART-D test scores.

These findings indicate that APT induces both a near transfer effect in the domain of the planning strategies and a far transfer effect in the domain of visuo-spatial memory.

Interestingly a previous RCT [18], which assessed the efficacy of APT in adult MS, found a significant improvement exclusively on tasks of sustained attention such as the PASAT and, marginally, the SDMT. No effect was detected on tests tapping other aspects of attention or other cognitive domains. The different effect of APT in POMS and adult MS provides support for the idea that restorative exercises started during the developmental age can induce a greater strengthening of the key brain networks implicated in attention processing. This can be explained by a greater brain plasticity in younger patients. Indeed, recent RCTs focused on attention rehabilitation and assessing fMRI outcomes demonstrated that intensive ST improved some aspects of cognitive functioning and also affected neural plasticity and increased fMRI brain activity in the cerebellum of adult MS patients with impaired attention and/or memory [19, 49].

Therefore, early detection and management of cognitive dysfunction is momentous in POMS.

Longitudinal studies in patients with POMS have provided conflicting results about the evolution of the cognitive deficits. Most of them [10, 11] have shown a stability in cognitive performance in POMS over time suggesting a lack of the expected age-related cognitive development. An Italian study lasting over 5 years demonstrated that cognitive deficits, mainly in visual-spatial learning and expressive language, tend to worsen over time, affecting the patient’s academic and professional achievements [13, 50].

Different studies have provided some information about the predictive factors associated to the longitudinal evolution of the cognitive performances in patients with POMS. Till C and colleagues reported that a longer disease duration was associated with greater deterioration in visuomotor integration and an increased lesion volume was associated with slower psychomotor speed over a 1 year follow-up [11]. Recently, Pastò and colleagues reported that the major predictor of cognitive stability or improvement over a follow-up longer than 4 years in patients with POMS was a higher cognitive reserve [51].

The availability of an easy to use and home-based cognitive training is of paramount importance in this pediatric population. In this light, another recent study investigated the feasibility of a home-based computerized program for working memory training in patients with POMS [52] supporting the use of this kind of approach to treat cognitive dysfunction in this specific population. Moreover, given all these premises, a computerized cognitive training would be recommended for all the patients with POMS as a tool to maintain level of cognitive functioning (i.e. before impairment becomes apparent) and to increase the cognitive reserve.

Referring to the ADHD population, our results show that APT is effective in improving the targeted cognitive domains which has been specifically trained by the program. In this population, APT determines only a slight improvement of the global cognitive functions, as measured by the reduction of the CII. These findings indicate that there is a less pronounced transfer effect in ADHD patients in comparison to POMS patients after the exposure to a cognitive training with a focus on attention. Although the different effect of the cognitive training in the two disease groups may be due to the more severe baseline cognitive impairment in ADHD patients, these results seem to be consistent with the majority of previous studies on cognitive rehabilitation which trains attention in ADHD patients [22, 28, 30, 53]. In these studies it has been show an improvement of the targeted ability with only a limited transfer to other cognitive performances. As a matter of fact, by considering attention as the key impairment in ADHD, the main selection criteria in such studies were based on the central-deficit assumption [29]. Moreover, the theoretical premise of these studies was that the remediation of attention deficits could reduce ADHD associated cognitive and behavioral difficulties [28, 30, 33, 53]. There is no a specific profile of executive functions impairment in ADHD [54].

Therefore, it is necessary to find out other approaches focusing on multiple neuropsychological processes to optimize the transfer of effect from cognitive deficit to clinical symptoms in ADHD.

It is noteworthy that in our study design we have also included a comparative training based on a nonspecific reinforce of text reading, comprehension and verbal performances that could have had an impact on the global cognitive functions [55, 56]. This could explain why in the nST group we have observed an improvement of the CII, although not statistically significant. These results are in line with previous findings in adult onset MS treated with the same cognitive training [18].

Several limitations of this study deserve discussion. First, the inclusion of two very diverse patient populations (POMS and ADHD), with small sample sizes in each group, may limit the generalizability of our findings. Secondly, the mean EDSS score level of our POMS cohort is slightly higher than that previously reported in other cohorts. This was due to the inclusion criteria which required only the enrollment of patients with a discrete cognitive deficit (with impairment in at least 2/4 attention) and thus with a slightly more severe course. Thirdly, we did not measure effort assessment (e.g., symptom validity measures), and most importantly we did not include measures of “clinically meaningful change” such as (e.g., school performance or parent reported behaviors) in the study protocol. Fourthly, we have applied the same cognitive battery to two different disease groups: POMS and ADHD. It is possible that the outcome assessments performed during the RCT were not sensitive to deficits in ADHD, but we choose the outcomes more recommended in POMS (at least in Italy and Europe) [9, 12, 13] and for a better comparison between the two groups, we used the same outcome assessments also in ADHD patients.

Referring to the statistical analysis, we should mention that we did not perform a multiple comparisons correction, therefore there is a potential risk of false positive results. We did so because we don’t think that the statistical approach of adjusting for multiple testing is necessary or even adequate. Reducing the type I error for null associations increases the type II error for those associations that are not null.

Finally, another limitation of this study should be discussed. The possibility that the positive effect of the ST we have observed is at least in part due to an expectancy effects among participants assigned to the ST.

However, this pilot study was designed for a fast evaluation of the efficacy of a cognitive training (in treated vs untreated patients) in the two different groups of pediatric patients (POMS or ADHD) with attention impairment. The internal validity and the consistency of the results are assured by a randomized controlled design and robust statistical analysis, which in part might mitigate all these limitations.

## Conclusions

Our results indicate that a cognitive rehabilitation program that targets attention may be a suitable tool for improving global cognitive functioning in POMS patients, whereas it has a less pronounced transfer effect in ADHD patients. Future RCTs on larger populations of both POMS and ADHD patients, including a healthy subjects control group, clinical and fMRI outcomes, are needed to confirm the efficacy of cognitive rehabilitation programs. Furthermore, future studies aimed to evaluate the efficacy of computerized cognitive rehabilitation program on remediating attention deficits should include a more specific sustained attention task as efficacy measure. Studies aimed at identifying factors that influence transfer effects, as well the mechanism underlying these effects, especially in ADHD patients and in subpopulations of POMS with and without ADHD symptoms are desirable.

## Abbreviations

ADHD: Attention Deficit Hyperactivity Disorder
CDI: Children’s Depression Inventory
nST: Non specific training
POMS: Pediatric onset multiple sclerosis
SDMT: Symbol Digit Modalities Test
SPART: Spatial Recall Test
SPART-D: Spatial Recall Test–Delayed
SRT-CTLR: Selective Reminding Test – Consistent Long-Term Retrieval
SRT-D: Selective Reminding Test–Delayed
SRT-LTS: Selective Reminding Test Long Term Storage
ST: Specific training
